# Longitudinal study of root resorption on incisors caused by impacted maxillary canines—a clinical and cone beam CT assessment

**DOI:** 10.1093/ejo/cjae052

**Published:** 2024-10-16

**Authors:** Anna Dahlén, Cecilia Persson, Sara Lofthag Hansen, Julia Naoumova

**Affiliations:** Department of Orthodontics, Institute of Odontology, Sahlgrenska Academy, University of Gothenburg, 413 90, Göteborg, Sweden; Clinic for Orthodontics, Gothenburg, Public Dental Service, Region Västra Götaland, Göteborg, Sweden; Clinic of Oral & Maxillofacial Radiology, Gothenburg, Public Dental Service, Region Västra Götaland, 413 90, Göteborg, Sweden; Clinic of Oral & Maxillofacial Radiology, Gothenburg, Public Dental Service, Region Västra Götaland, 413 90, Göteborg, Sweden; Department of Orthodontics, Institute of Odontology, Sahlgrenska Academy, University of Gothenburg, 413 90, Göteborg, Sweden; Clinic for Orthodontics, Gothenburg, Public Dental Service, Region Västra Götaland, Göteborg, Sweden

**Keywords:** impacted maxillary canines, maxillary resorbed incisors, root resorption, resorption grade, long-term follow-up, CBCT

## Abstract

**Objective:**

To evaluate the long-term status of incisors with canine-induced root resorption (CIRR).

**Materials and methods:**

Subjects with impacted maxillary canines (IMC) and persisting incisors with CIRR examined with cone beam computed tomography (CBCT), diagnosed ≥ 5 years earlier, were recalled. The resorption grade in the horizontal and vertical plane was assessed on CBCT images at baseline (T0) and follow-up (T1). Clinical examination was done at T1 which included probing depth, gingival retraction, mobility, ankylosis, discoloration and vitality test. In addition, patients completed a questionnaire regarding symptoms from the incisors.

**Results:**

Forty subjects (mean age 13.7 ± 2.1 years) with 43 IMC and 47 incisors with CIRR were recruited. The IMC either spontaneously erupted, were surgically exposed or surgically removed. Thirty-four of the patients were treated with a fixed appliance and six had no orthodontic treatment. The follow-up range was 5.5–14.6 years. None of the incisors were lost or endodontically treated at T1. The horizontal resorption grade was unchanged in 38, improved in 7, and worsened in 2 teeth. The corresponding results for the vertical resorption grade were unchanged in 20 and worsened in 27 teeth. Three incisors with severe horizontal resorption at T0 were significantly more obliterated at T1 (*P* = .01). No significant differences were found in clinical parameters or patient-reported outcomes between incisors with CIRR and non-resorbed contralateral incisors at T1.

**Limitations:**

The extent of root resorption during active orthodontic treatment was not possible to assess as only CBCT images from T0 and T1 were available.

**Conclusion:**

Incisors with CIRR caused by IMC have a high survival rate in a long-term perspective and do not cause more symptoms or exhibit more signs of pathology than non-resorbed contralateral incisors. Extraction of asymptomatic incisors based solely on root resorption should not be performed routinely.

## Introduction

After the third molar, the maxillary canine is the most frequently displaced tooth with a prevalence ranging from 1.1% to 3.3% [1–5] and is more common in females [3, 6]. Root resorption of adjacent incisors is a well-known complication of impacted maxillary canines (IMC) [7, 8]. The incidence varies widely between different studies and ranges from 25% to 67% for lateral incisors and 5%–31% for central incisors [6, 9–12]. Teeth with root resorption, even with severe lesions and pulp involvement, are in most cases asymptomatic [13–15]. Radiographic examination is therefore necessary in cases where ectopic eruption cannot be dismissed by a clinical examination [16]. The degree of root resorption is often considered an important factor that determines the orthodontist’s therapy choice [17]. If root resorption is suspected, three-dimensional radiographic imaging is superior to conventional radiographs and is sometimes indicated in identifying and assessing the extent of the resorption [18–23]. The use of cone beam computed tomography (CBCT) is thought to affect the orthodontist’s confidence level in a positive direction [24, 25] and may alter the treatment plan compared with only two-dimensional radiographs [18, 25, 26].

Many studies have aimed at identifying factors that predict which canines induce root resorption; however, without reaching a consensus [11, 27–29]. One of the latest systematic reviews concluded that root resorption of maxillary incisors was correlated with their direct contact with the erupting canines. No other significant factors related to root resorption were identified [7]. According to another systematic review, there is little evidence of the effectiveness of various interventions to manage resorption. The clinicians should therefore base their therapy choice on the clinical experience together with the patients’ preferences [30]. Extraction of severely resorbed incisors has sometimes been the choice of therapy because of the assumption of a poor long-term prognosis [31].

There are only a few long-term follow-up articles available in the literature evaluating the clinical and radiographic status of resorbed maxillary incisors caused by IMC. The survival rate is, however, reported to be high (93%–100%), mobility and ankylosis are rare and most of the root resorptions are unchanged or improved compared with baseline. The long-term prognosis is therefore suggested to be good [32–34]. However, the follow-up period in these studies had a wide range from 1 to 28 years and none included patients with CBCT examinations both at baseline and at follow-up. In addition, they did not include patient-reported outcomes or clinical examinations for all patients at the follow-up stage, except Falahat *et al*. [34], but their assessment was limited to vitality and percussion tests. To our knowledge, there are no studies in the literature with computed tomography (CT) or CBCT before and after treatment of non-erupted/impacted canines, assessing resorbed maxillary incisors over time combined with a clinical examination and patient-reported outcome. Therefore, in this long-term follow-up study, the primary aim was:

- To evaluate the survival rate of maxillary resorbed incisors.

The secondary aims were:

- To compare the extent of root resorption before any interventions and at the long-term follow-up.
- To investigate whether the resorbed incisors have deviant clinical findings in the long-term perspective.
- To assess whether the patient has symptoms from the affected incisors.

## Materials and methods

### Sample selection

Data for this retrospective cohort study were collected from a pool of available CBCT records of patients referred to the Specialist Clinic of Oral and Maxillofacial Radiology, Gothenburg, Sweden, the Specialist Clinic of Orthodontics and the Specialist Clinic of Pedodontics in Gothenburg and Mölndal, Sweden, between 2005 and 2015. Patients with IMC examined with CBCT and diagnosed with root resorption of at least one incisor were selected for further analysis.

The final sample was selected based on the following eligibility criteria:

#### Inclusion criteria

**Figure 1.**
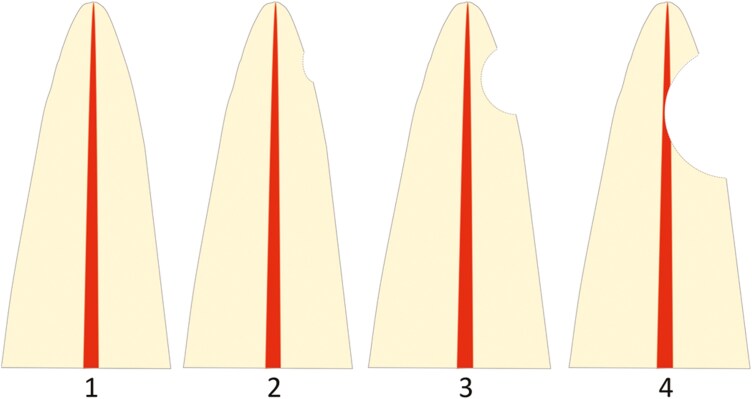
Horizontal resorption according to the index suggested by Ericsson and Kurol [19]: (1) no resorption, intact root surfaces, and the cementum layer may be lost; (2) slight resorption, resorption up to half of the dentine thickness to the pulp; (3) moderate resorption, resorption midway to the pulp or more, the pulp lining being unbroken; (4) severe resorption, the pulp is exposed by the resorption.

- Unilateral or bilateral IMC.
- One or more resorbed maxillary incisor/s (classified as grades 2–4 according to the index suggested by Ericsson and Kurol [19] (Fig. 1), that were not extracted.
- Baseline CBCT examination, when impaction was diagnosed, performed at least five years prior to the follow-up.

#### Exclusion criteria

- Cleft lip and palate and craniofacial syndromes.
- Previous orthodontic treatment performed before the baseline CBCT examination.

### Registration

This trial was registered in “FoU i Sverige” (Research and Development in Sweden), registration number: 941656.

### Ethical issue

The study was preapproved by the Swedish Ethical Review Authority (Reg. no. 2020-06519). All eligible patients who could be contacted received verbal and written information and were asked to sign the informed consent form before entering the trial.

### Questionnaire and examination

Patients accepted to participate in this follow-up study completed a questionnaire and underwent a clinical and radiographic examination at the same appointment. CBCT in orthodontics should be used according to the ALADA-principle (as low as diagnostically acceptable) [35]. However, the second CBCT examination in this study was justified by the fact that three-dimensional radiographic imaging is superior to conventional radiographs in diagnosing root resorption [20] and to examine how resorption lesions develop over time the baseline images had to be compared with the follow-up CBCT-images.

#### Questionnaire

The questionnaire included general questions about the patient’s health and whether they had any symptoms from their anterior maxillary teeth. Details are given in Appendix 1.

#### Clinical examination

Clinical examinations were performed by two calibrated operators (AD and JN). The following clinical parameters were recorded on all central and lateral maxillary incisors: survival rate (defined as; the presence of the resorbed incisor at the follow-up), probing depth, gingival retraction, mobility, ankylosis, discoloration and vitality.

Information was obtained from the dental records about which intervention was performed for the IMC. The clinical examination is described in detail in Appendix 2.

#### Radiographic examination

The CBCT machine used for the radiographic examination at baseline (T0) and follow-up (T1) was a Morita Accitomo^®^. At T0, the operating parameters were 80, 85, or 90 kVp, 5–6 mA with rotations of 180° or 360°, while at T1, the operating parameters were 90 kVp, 5–6 mA with a rotation of 180°. The same field of view (4 × 4 cm) and slice thickness (0.48000 mm) were used at T0 and T1. The examinations were sent to Sectra PACS^®^ and multiplanar reconstruction (MPR) along the long axis of the resorbed incisor was created with the image distance set to 0.5 mm and the slice thickness to 0.5 mm. The cusp position of the IMC in the buccopalatal plane was assessed as central, palatal, or buccal location in the dental arch. The lateral and central incisors were analyzed for resorption in the axial, cross-sectional, and sagittal plane. The following radiographic parameters of the incisors with canine-induced root resorption (CIRR) were analyzed at T0 and T1:

##### Horizontal resorptions:

measured at its most severe site and graded according to the index suggested by Ericsson and Kurol [19] (Fig. 1).

##### Vertical resorptions:

**Figure 2.**
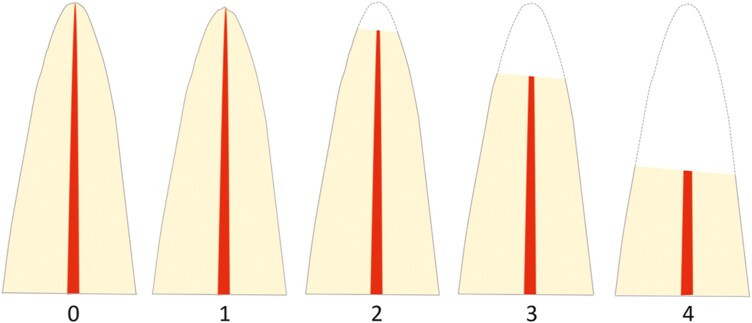
Vertical resorptions graded according to the index suggested by Malmgren [36]: (0) no resorption; (1) irregular apical root surface; (2) apical resorption < 2 mm; (3) apical resorption from 2 mm and up to 1/3 of the original root length; (4) apical resorption > 1/3 of the original root length.

measured from the cementoenamel junction to the most apical part of the root and graded according to the index by Malmgren [36] (Fig. 2). In 15 patients, the non-resorbed contralateral incisor was visible at T0, and the vertical dimensions of these incisors were also assessed at both T0 and T1.

##### Resorption surface:

registered as buccal, mesial, palatal, and distal and divided into five groups: buccal/distobuccal, palatal/distopalatal, distal, mesial, and apical. Apical resorption indicates resorption at all four surfaces at the apical tip of the incisor.

##### Localization of the resorption:

registered as cervical, middle, or apical third of the root.

##### Pulp canal obliteration (PCO):

the diameter of the pulp canal is measured in the axial plane at its narrowest part and compared between T1 and T0. Changes <1.0 mm were not registered.

The radiological parameters were assessed by two calibrated specialists in oral maxillofacial radiology (SLH and CP). Linear measurements were measured to the closest 0.1 mm using an Eizo (black and white) screen. To assess intra-examiner reliability, ten images (21%) were randomly selected and remeasured.

#### Blinding

During the clinical examination, the operators were blinded to which teeth were diagnosed with root resorption. The radiographic assessment at T1 was blinded to previous assessments and measured resorption at T0.

### Statistical analysis

Data were imported to SAS (Version 9.1) for statistical analysis. Descriptive statistics and arithmetic means and standard deviations were measured for numerical variables.

A comparison of numerical data between the radiographic indices and differences in grade of resorption was performed with Fisher’s exact test. The effect of time variables on changes in horizontal root resorption was assessed using a one-way ANOVA test and a paired *t*-test was used for vertical root resorption. The results were reported at patient and tooth level. The level of statistical significance was set to 0.05. Intra-examiner reliability was examined with the Cohen kappa test. For horizontal resorption the intra-examiner reliability was 0.8 and for vertical resorption 0.7, indicating excellent to good strength of agreement.

## Results

Ninety-nine subjects with at least one incisor with CIRR grades 2–4 were identified, 36 of these were excluded because the affected incisor was extracted. The excluded subjects had 49 incisors with CIRR: nine with grade 2, 14 with grade 3, and 26 with grade 4. Significant more incisors with grade 4 and significantly fewer incisors with grade 2 resorption were extracted compared to the eligibility subjects.

**Figure 3.**
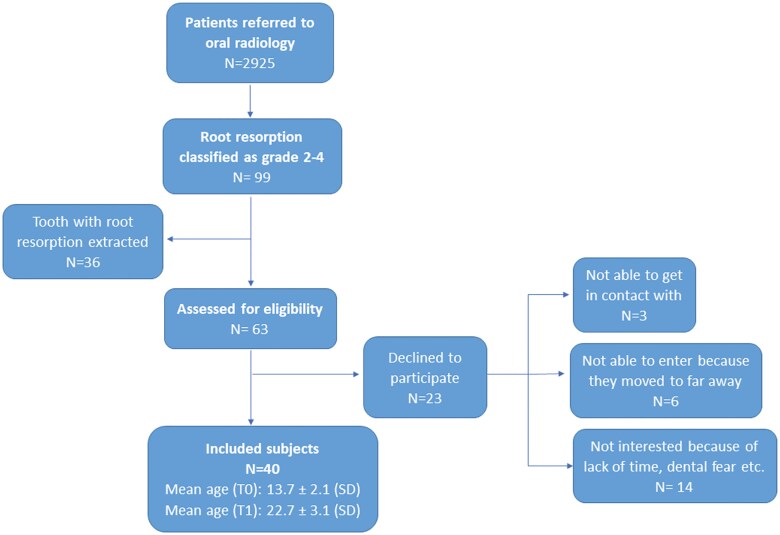
Flowchart of sample selection. Mean age in years at T0 = baseline, T1 = follow-up.

A total of 40 patients were included, 34 females and six males (Fig. 3) with a long-term follow-up ranging from 5.5 to 14.6 years (mean 9.1 ± 2.5 SD). Forty-three IMCs and 47 incisors with CIRR (41 laterals and six centrals) were reexamined at T1. Twenty-seven of the IMCs were unilateral and 13 bilateral but only three of the bilateral IMCs had caused root resorption on both sides. Therefore, a non-resorbed contralateral incisor was present in 41 of the incisors with CIRR. Bilateral root resorption was diagnosed in the remaining six incisors.

In two patients the IMCs erupted spontaneously, 23 were treated with surgical exposure and 15 with surgical removal. In one patient the intervention was performed prior to T0. In the remaining 37 patients, the interventions were performed 5.8 ± 5.6 (SD) months after T0. Twenty-two of the IMCs were positioned palatally, five centrally and 15 buccally. Thirty-four of the patients were treated with a fixed appliance and six had no orthodontic treatment. One patient ended the treatment in another country and therefore data of treatment time is missing. The 33 remaining patients had a mean treatment time of 25.6 ± 8.8 months.

### Questionnaire results

No statistically significant differences were found between incisors with CIRR and non-resorbed contralateral incisors with regard to tenderness (*n* = 1 and *n* = 2, respectively), pain (*n* = 2 and *n* = 2), sensitivity to cold/warm drinks (*n* = 12 and *n* = 9) or deviant color (*n* = 2 and *n* = 2).

None of the patients had been treated with radiation therapy. Two patients reported trauma to teeth, these had composite restorations on one of their central incisors, but the teeth with root resorption were in both cases lateral incisors. Fifteen patients reported a known allergy, with pollen being the most common allergen. Patients with allergy did not have a statistically higher resorption grade at T0 or T1.

### Clinical results

**Figure 4.**
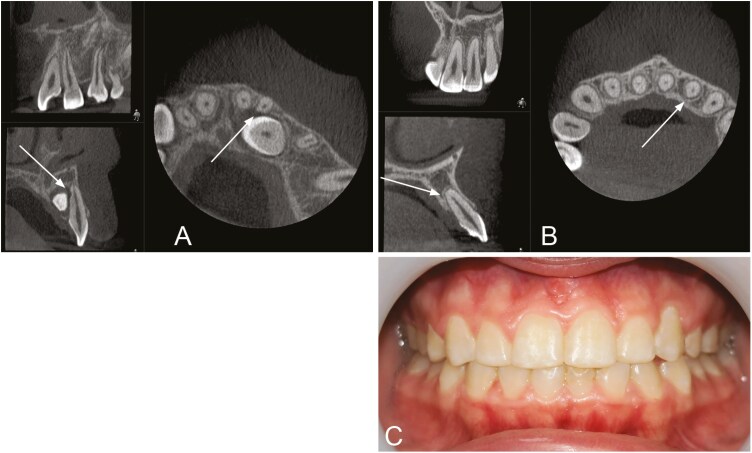
Tooth 22 exhibits horizontal resorption grade 3 at T0 (A) and grade 2 at T1 (B). The vertical resorption grade was 2 at both T0 and T1. The patient had no symptoms or deviant clinical findings (C).

**Figure 5.**
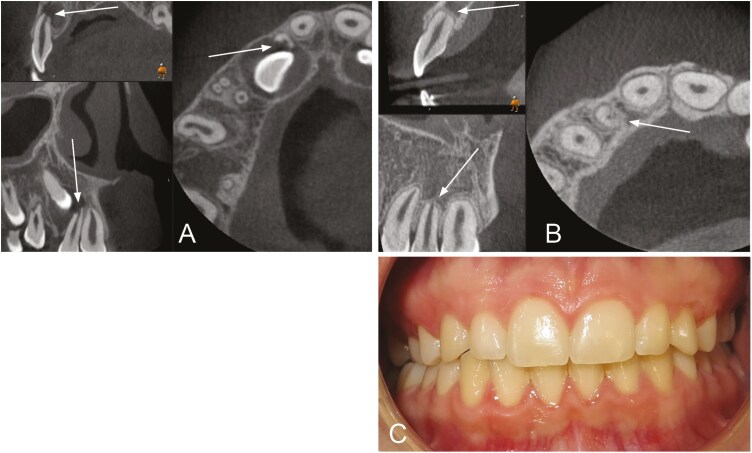
Tooth 12 exhibits horizontal resorption grade 4 at both T0 (A) and T1 (B). The vertical resorption was grade 2 at T0 and grade 4 at T1. The patient had no symptoms or deviant clinical findings (C).

**Figure 6.**
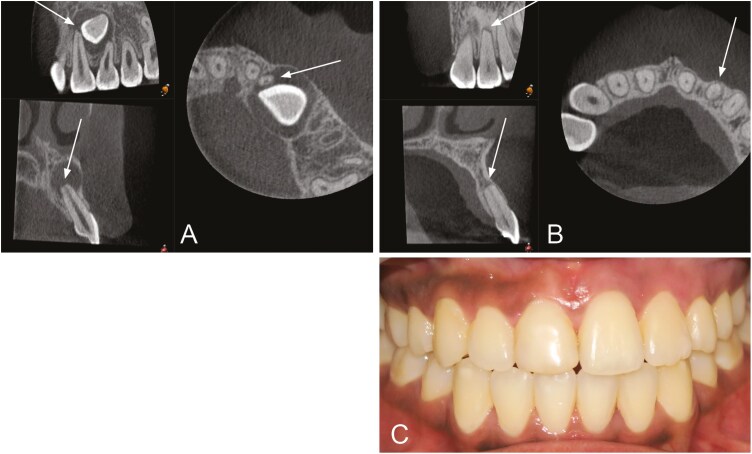
Tooth 22 exhibits horizontal resorption grade 2 at T0 (A) and grade 3 at T1 (B). The vertical resorption grade was grade 2 at T0 (A) and grade 3 at T1 (B). Obliteration of the pulp was seen at T1 (B), and the tooth did not respond to electric pulp testing. The patient had no symptoms (C).

None of the 47 resorbed incisors had been extracted, giving a 100% survival rate. In addition, none of the teeth had undergone root canal treatment during the follow-up period. There were no significant differences between incisors with CIRR and non-resorbed contralateral incisors regarding discoloration, mobility, ankylosis, gingival retraction or vitality (Figs. 4–6). Non-resorbed incisors had a statistically significantly higher incidence of gingival pockets with a probing depth ≥ 4 mm compared with incisors with CIRR (*P* = .02). No incisors had pockets with a probing depth greater than 5 mm (Table 1).

**Table 1.** Clinical findings at follow-up in incisors with canine-induced root resorption (CIRR) and non-resorbed contralateral incisors.

	Incisors with CIRR (%)			Non-resorbed contralateral incisors (%)			*P* value
	Lateral (*n* = 41)	Central (*n* = 6)	Total (*n* = 47)	Lateral (*n* = 35)	Central (*n* = 6)	Total^a^ (*n* = 41)	
Discoloration^b^	0 (0)	0 (0)	0 (0)	0 (0)	0 (0)	0 (0)	*P* > .05
Probing depth ≥ 4 mm	2 (5)	0 (0)	2 (4)	6 (17)	0 (0)	6 (15)	***P* < .02**
Increased mobility	3 (7)	0 (0)	3 (6)	1 (3)	0 (0)	1 (2)	*P* > .05
Ankylosis	0 (0)	0 (0)	0 (0)	0 (0)	0 (0)	0 (0)	*P* > .05
Gingival retraction	3 (7)	0 (0)	3 (6)	2 (6)	0 (0)	2 (5)	*P* > .05
No vitality	1 (2)	0 (0)	1 (2)	0 (0)	0 (0)	0 (0)	*P* > .05

Bold values indicate *P* = .0183.

^a^Six patients had resorption on both lateral incisors and therefore no non-resorbed contralateral incisors.

^b^Assessed by the clinician and defined as too yellow, gray, dark, or ligh.

There were no significant differences between incisors with a higher grade of vertical and/or horizontal resorption with respect to discoloration, probing depth, mobility, ankylosis, gingival retraction, or vitality (Table 2). Resorbed incisors with resorption extending into the dental pulp did not show more clinical signs of pathology than incisors with only slight or no signs of resorption.

**Table 2.** Clinical findings of the 47 incisors with canine-induced root resorption (CIRR) at follow-up divided into horizontal and vertical resorption grade.

	Horizontal resorption grade (*n* = 47)				Total	Vertical resorption grade (*n* = 47)					Total
	1	2	3	4		0	1	2	3	4	
Discoloration^a^	0	0	0	0	0	0	0	0	0	0	0
Probing depth ≥ 4 mm^b^	0	0	0	2	2	0	0	1	1	0	2
Increased mobility	0	1	0	2	3	1	0	1	0	1	3
Ankylosis	0	0	0	0	0	0	0	0	0	0	0
Gingival retraction	1	1	0	1	3	2	0	0	1	0	3
No vitality	0	0	0	1	1	0	0	0	1	0	1

^a^Assessed by the clinician and defined as too yellow, gray, dark, or ligh.

^b^On at least one site.

### Radiographic result

#### Localization

Resorptions were mostly found in the middle of the root at T0 and in the apical third of the root at T1. Palatal/distopalatal and buccal/distobuccal surfaces were mostly affected at both T0 and T1 (Table 3). Figs. 4–6 show examples of patients with horizontal and vertical resorption grades at T0 and T1.

**Table 3.** Localization of resorption lesion and horizontal and vertical root resorption at baseline (T0) and follow-up (T1) on incisors with canine-induced root resorption (CIRR).

	Baseline (T0)			Follow-up (T1)		
	Lateral (*n* = 41)	Central (*n* = 6)	Total (*n* = 47) (%)	Lateral (*n* = 41)	Central (*n* = 6)	Total (*n* = 47) (%)
**Localization of the resorption**						
Cervical third	7	1	8	9	2	11
Middle third	20	4	24	10	3	13
Apical third	14	1	15	22	1	23
**Resorbed surfaces** ^a^						
Buccal, distobuccal	15	0	15	14	4	18
Palatal, distopalatal	16	4	20	20	0	20
Distal	5	2	7	2	1	3
Mesial	6	1	7	1	0	1
Apical	6	0	6	6	0	6
**Root resorption horizontal**						
Grade 1	0	0	0 (0)	1	2	3 (6)
Grade 2	14	4	18 (38)	15	2	17 (36)
Grade 3	16	1	17 (36)	14	1	15 (32)
Grade 4	11	1	12 (26)	11	1	12 (26)
**Root resorption vertical**						
Grade 0	18	5	23 (49)	11	1	12 (26)
Grade 1	8	0	8 (17)	5	3	8 (17)
Grade 2	9	0	9 (19)	11	1	12 (26)
Grade 3	5	1	6 (13)	8	0	8 (17)
Grade 4	1	0	1 (2)	6	1	7 (15)

^a^On some incisors, the resorption affected more than one surface; therefore, the total number of resorbed surfaces exceeds the total number of incisors.

#### Resorption changes over time

**Figure 7.**
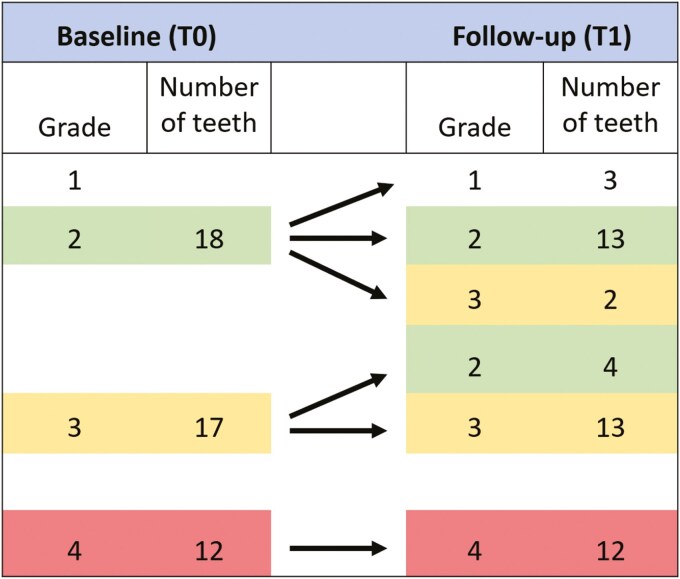
Changes in horizontal resorption grade between baseline (T0) and follow-up (T1) on incisors with CIRR.

**Figure 8.**
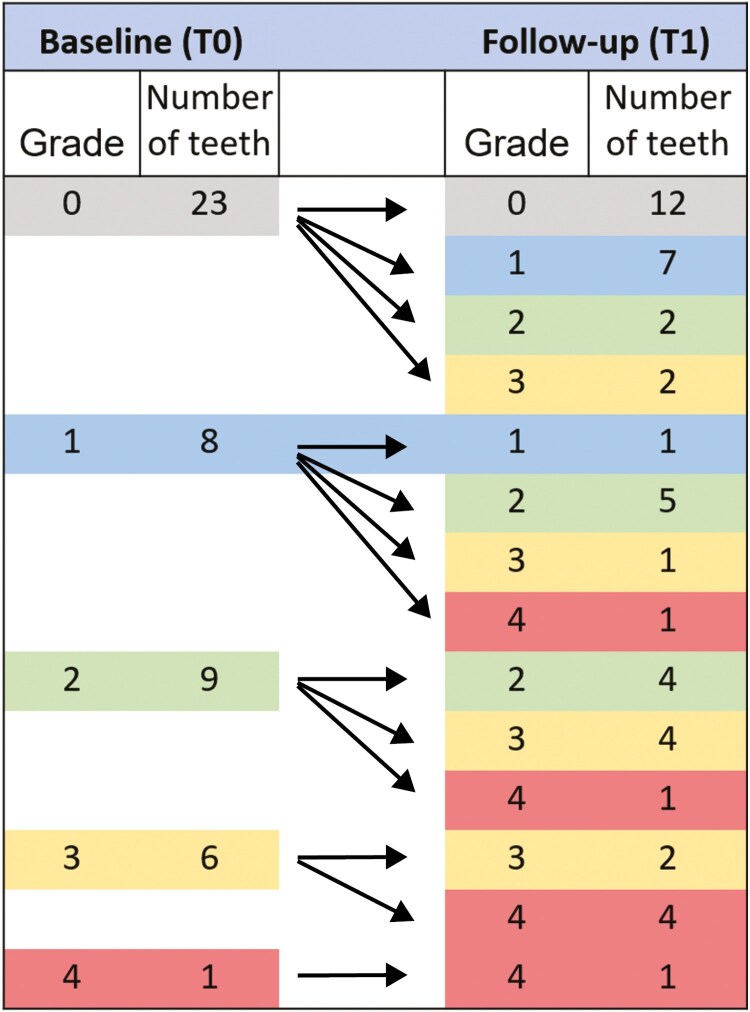
Change in vertical resorption grade between (baseline) T0 and (follow-up) T1 on incisors with CIRR.

The incidence of incisors with different horizontal resorption grades was unchanged from T0 to T1 in 80.9% of the incisors with CIRR, improved in 14.9% and worsened in 4.3% (Table 3, Fig. 7). The incidence of incisors with different vertical resorption grades was unchanged in 42.6% of the incisors with CIRRs and worsened in 57.4% during the follow-up period (Table 3, Fig. 8). The change in horizontal and vertical resorption grade from T0 to T1, relative to the grade at T0, was not statistically significant.

In the 15 non-resorbed contralateral incisors that were visible on the CBCT examination at both T0 and T1, the incidence of vertical resorption was unchanged in 40.0% of the incisors and increased in 60.0%. The distribution of the grades was: grade 2: 46.7%, grade 3: 6.7% and grade 4: 0%. There was no statistically significant difference in the changes in vertical resorption from T0 to T1 between the incisors with CIRR and non-resorbed contralateral incisors (Table 4).

**Table 4.** Change in vertical resorption from baseline (T0) to follow-up (T1) in 15 incisors with canine-induced root resorption (CIRR) and the corresponding non-resorbed contralateral incisors.

	Incisors with (CIRR) (*n* = 15)	Non-resorbed contralateral incisors (*n* = 15)	*P* value
Unchanged	9	6	*P* > .05
Worsened	6	9	*P* > .05

There was no statistically significant association between the changes in either the horizontal or vertical resorption and the time frame for diagnosing the resorption until the intervention for the IMC took place, the treatment duration with fixed appliance, or the duration of the follow-up period (Supplementary Table 1).

#### Side-effects of the resorption

Three patients had one incisor each with pulp canal obliteration (PCO) at T1, which was not seen at T0. The incisors with CIRR with PCO all had grade 4 horizontal resorption, which was statistically significantly different compared to those with grades 1-3 where no PCO was seen (*P* = .01). None of the non-resorbed contralateral incisors had PCO.

## Discussion

There are only a few long-term follow-up articles assessing the prognosis of resorbed incisors caused by IMC [32–34]. The objective of this longitudinal cohort study was therefore to evaluate the survival rate of incisors with CIRR and whether these teeth have deviant clinical and radiographic findings or symptoms. The results showed that the affected incisors had a 100 per cent survival rate and did not cause any deviant clinical findings or symptoms compared with the unaffected anterior teeth.

The absence of symptoms and pathologies found in the clinical examination in the present study agrees with previous findings [32–34]. Despite significantly more non-resorbed contralateral incisors having a probing depth ≥ 4 mm, it was not considered clinically relevant as, in total, there were only six non-resorbed contralateral incisors and two incisors with CIRR with 4 or 5 mm of probing depth.

Of all the incisors with CIRR, only one exhibited a negative response to a vitality test (Fig. 6). This incisor was, however, asymptomatic, displayed no signs of an apical pathology and possessed a normal lamina dura, but had pulp canal obliteration (PCO). PCO is a well-known biological reaction to dental trauma and can under certain circumstances develop into a necrotic pulp [37]. Previous studies have observed PCO in incisors with CIRR [32–34] and reported that there is an association with orthodontic treatment and obliteration [38]. In the current study, three of the incisors with CIRR had PCO. These patients were treated with fixed appliances, but since no radiological examination was made after completion of the orthodontic treatment, we could not determine when the PCO had developed. PCO was found significantly more often in incisors with horizontal resorption grade 4 than grades 1–3. However, this result should be considered with caution due to the limited number of incisors with PCO. Further research is necessary to explore if there is a correlation between severe root resorption and PCO.

Allergies were reported since they have been positively correlated in previous studies with a high root resorption response to orthodontic forces; however, without being statistically significant [39]. In this study, 32% of the patients reported allergies and these patients did not have a significantly greater degree of horizontal or vertical root resorption.

Most of the incisors had no change in the horizontal resorption grade between T0 and T1. This finding supports the conclusion that once the impacted canine has been treated; i.e. the pressure from the IMC eliminated, there is no risk of further resorption [32]. Case reports have also indicated that the resorptive process ceases when the IMC inducing the resorption is removed [40, 41]. Only two patients in this study had an increased horizontal resorption grade at T1 compared with T0. An explanation for this could be the prolonged time the canine persisted in the ectopic position. One of these patients had the longest time span between diagnosis and intervention as the exposure was made 20 months after the CBCT image was taken. The other patient was first treated with surgical exposure and a year later with surgical extraction since no improvement of the canine position was seen.

An improvement of the horizontal grade at T1 was observed in seven incisors, which might be explained by the smothering of the root surface, remodeling and formation of new root cementum that occurs following the removal of an IMC [42, 43]. In addition, using indices to evaluate changes in root resorption has its limitations as the assessment is based on the dentin width in relation to the pulp and a narrower pulp at T1 will result in a less severe resorption grade, even if the amount of dentin or cementum in the resorption lesion is unchanged. The sign of improvement of the root resorption is in accordance with the results from other studies [33, 34].

Root resorption is a common side effect of orthodontic treatment affecting 79%–94% of the patients, according to histological and CT studies [44–46], while studies with intraoral radiographic assessment report 50%–66% [47, 48]. The maxillary incisors are the most affected teeth [46]. Longitudinal studies indicate that after discontinuance of the orthodontic forces, the resorbing process does not progress and teeth with a healthy periodontal ligament remain stable, despite severe root resorption [49, 50]. In our study, the vertical resorption grade increased between T0 and T1, which was not surprising as 34 out of 40 patients were treated with a fixed appliance. The vertical resorption grade did not, however, increase significantly more in the incisors with CIRR compared with the non-resorbed contralateral incisors, indicating that resorption caused by IMC does not affect the root resorption caused by the orthodontic treatment. Levander *et al*. reported that grade 2 vertical root resorption occurs in 48%, grade 3 in 17% and grade 4 in 1% of the incisors after orthodontic treatment [47]. These numbers are similar to our results regarding the non-resorbed contralateral incisors. The incisors with CIRR, on the other hand, had less grade 2 resorption (26%), similar numbers with grade 3 (17%) and more resorptions with grade 4 (15%). This difference could be explained by the fact that the horizontal root resorption was located in the apical third of the incisor root in about 50% of the IMC and the teeth were thus affected by resorption already when the orthodontic treatment started.

Surgical exposure of the IMC followed by orthodontic traction was performed in 23 out of 40 subjects. It has been suggested that orthodontic traction after surgical exposure does not worsen incisor root resorption [51].

### Limitations and strengths

CBCT has only been used for this patient group since the mid-2000s and teeth with more severe root resorption are/were often extracted due to an uncertain prognosis, which could have added some selection bias and limited our ability to gather a large group of patients for the study. However, previous research indicates that significantly more root resorptions are diagnosed when CBCT is used compared with conventional apical radiography [26]. This suggests that a larger number of incisors were left untreated before the widespread adoption of CBCT, as root resorption was not detected. This study shows that the prognosis is good, even for severely resorbed incisors. The precise and detailed information provided by the CBCT does not necessarily benefit the patients as incisors with a good long-term prognosis might be extracted due to the clinician’s overestimation of the radiological findings in a negative direction.

The present study could not assess the amount of root resorption that was caused solely by the orthodontic treatment, due to the absence of radiographic images taken immediately after the treatment. However, a significant difference was not observed for vertical root resorption changes on incisors with CIRR compared with non-resorbed contralateral incisors.

The current study has notable strengths including CBCT before and after treatment, as three-dimensional radiographic imaging is superior to conventional radiographs in measuring the extent and the changes in root resorption lesions [20]. To minimize the radiation dose, we used a smaller field of view (4 × 4 cm). However, the result of this study shows that there is no need in clinical practice to follow-up root resorptions with a second CBCT since resorption lesions do not worsen over time. We recommend therefore to follow the ALADA principle [35]. Another strength of the study was the extensive clinical examinations and patient-reported outcomes that were collected for all subjects. Furthermore, data from the clinical assessment and the questionnaire were collected on all four incisors, but only outcomes from the incisors with CIRR and non-resorbed contralateral incisors were reported. The reason for this was the blinded data collection, as neither the patients nor the observer knew which tooth or teeth were resorbed. The resorption changes measured by volume will be presented in the coming article.

## Conclusions

Incisors with root resorption caused by IMC have a high survival rate. In addition, patients have few symptoms and negative clinical findings are rare. The process of root resorption does not appear to continue after the pressure from the IMC is removed. There is no evidence to suggest that incisors with CIRR are more prone to root resorption during orthodontic treatment. Finally, the findings of this study suggest that caution be observed when considering extraction of incisors based solely on root resorption and when interpreting the result of CBCT examinations.

## Supplementary Material

cjae052_suppl_Supplementary_Material

## Data Availability

Data underlying this publication will be shared on reasonable request to the corresponding author.

## Harms

Nothing to declare.
