# Correction to: Longitudinal study of root resorption on incisors caused by impacted maxillary canines—a clinical and cone beam CT assessment

**DOI:** 10.1093/ejo/cjaf090

**Published:** 2025-10-16

This is a correction to: Anna Dahlén, Cecilia Persson, Sara Lofthag Hansen, Julia Naoumova, Longitudinal study of root resorption on incisors caused by impacted maxillary canines—a clinical and cone beam CT assessment, *European Journal of Orthodontics*, Volume 46, Issue 6, December 2024, https://doi.org/10.1093/ejo/cjae052

The following changes have been made to the originally published manuscript.

Under **Resorption changes over time**, text is emended to read (differences are marked underlined and in bold type):

“The incidence of incisors with different horizontal resorption grades was unchanged from T0 to T1 in 80.9% of the incisors with CIRR, improved in 14.9% and worsened in 4.3% (Table 3, Fig. 7). The incidence of incisors with different vertical resorption grades was unchanged in 42.6% of the incisors with CIRRs and worsened in 57.4% during the follow-up period (Table 3, Fig. 8). **The change in horizontal and vertical resorption grade from T0 to T1, relative to the grade at T0, was not statistically significant**.” instead of:

“The incidence of incisors with different horizontal resorption grades was unchanged from T0 to T1 in 80.9% of the incisors with CIRR, improved in 14.9% and worsened in 4.3% (Table 3, Fig. 7). **However, these changes were not statistically significant**. The incidence of incisors with different vertical resorption grades was unchanged in 42.6% of the incisors with CIRRs and worsened in 57.4% **(*P* = .01)** during the follow-up period (Table 3, Fig. 8).”

Appendices 1 and 2 were missed from the original publication. These have now been added to the **Supplementary Data** section.

The emendations have been made to the article.

