# Supplementary material for: Longitudinal study of root resorption on incisors caused by impacted maxillary canines—a clinical and cone beam CT assessment
Source: Eur J Orthod. 2024 Oct 16;46(6):cjae052. doi: 10.1093/ejo/cjae052 (PMC11480922; doi:10.1093/ejo/cjae052)
Supplement: cjae052_suppl_Supplementary_Material [file cjae052_suppl_supplementary_material.zip › Appendix_1.docx]

QUESTIONNAIRE

Name………………………….......................................................

Date of birth: ………………………….…

1. Do you have any type of allergy or hypersensitivity?

Yes _________________________ No

If yes, to what:

1. Have you ever had any radiation therapy?

Yes _________________________ No

If yes, why:

1. Have you experienced a trauma or any violence to your face or teeth?

Yes _________________________ No

If yes, briefly describe what happened and when:

1. Does it feel strange / tender when biting with your front teeth in the upper jaw?
   - No

Yes, which tooth?

- - Large front tooth on the right side
  - Small front tooth on the right side
  - Large front tooth on the left side
  - Small front tooth on the left side
  - All front teeth/I don’t know which one

1. Do you have pain in any front tooth in the upper jaw?
   - No

If yes, which tooth?

- - Large front tooth on the right side
  - Small front tooth on the right side
  - Large front tooth on the left side
  - Small front tooth on the left side
  - All front teeth/I don’t know which one

1. Do you have any sensitivity of the front teeth in the upper jaw when consuming hot drinks?
   - No sensitivity

If yes, which tooth?

- - Large front tooth on the right side
  - Small front tooth on the right side
  - Large front tooth on the left side
  - Small front tooth on the left side
  - All front teeth/I don’t know which one

6. How much trouble, if any, do you experience with sensitivity on a scale of 1-10?

[
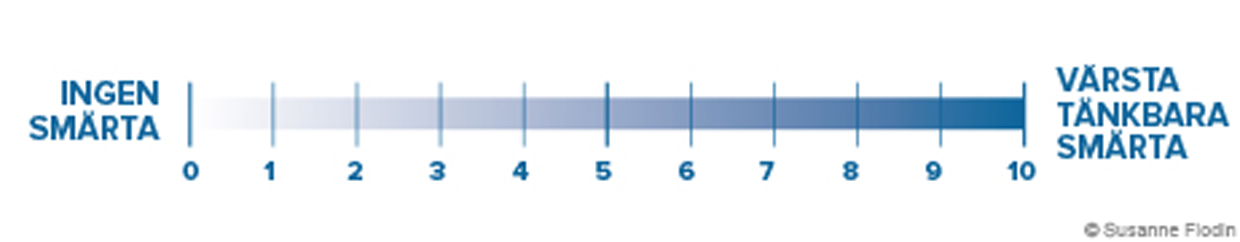
](https://www.google.se/url?sa=i&rct=j&q=&esrc=s&source=images&cd=&ved=2ahUKEwiS38XDlozjAhUQtIsKHR1hDcYQjRx6BAgBEAU&url=https://www.vardhandboken.se/vard-och-behandling/akut-bedomning-och-skattning/smartskattning-av-akut-och-postoperativ-smarta/smartskattningsinstrument/&psig=AOvVaw1F53y4AKmIkj7Sv-RoOtNp&ust=1561811124900184)

Not at all Extremely much

7. Do you have any sensitivity of the front teeth in the upper jaw when consuming cold drinks?

- - No, sensitivity

If yes, which tooth?

- - Large front tooth on the right side
  - Small front tooth on the right side
  - Large front tooth on the left side
  - Small front tooth on the left side
  - All front teeth/I don’t know which one

1. How much trouble, if any, do you experience with sensitivity on a scale of 1-10?

[
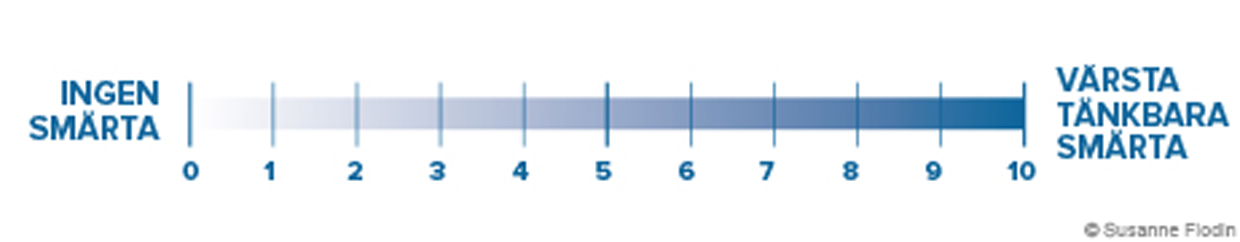
](https://www.google.se/url?sa=i&rct=j&q=&esrc=s&source=images&cd=&ved=2ahUKEwiS38XDlozjAhUQtIsKHR1hDcYQjRx6BAgBEAU&url=https://www.vardhandboken.se/vard-och-behandling/akut-bedomning-och-skattning/smartskattning-av-akut-och-postoperativ-smarta/smartskattningsinstrument/&psig=AOvVaw1F53y4AKmIkj7Sv-RoOtNp&ust=1561811124900184)

Not at all Extremely much

1. What do you think about the color of your front teeth in the upper jaw?

| Small front tooth on the right side | Large front tooth on the right side | Large front tooth on the left side | Small front tooth on the left side |
| --- | --- | --- | --- |
| - Too yellow - Too grey - Too dark - Too light - Good | - Too yellow - Too grey - Too dark - Too light - Good | - Too yellow - Too grey - Too dark - Too light - Good | - Too yellow - Too grey - Too dark - Too light - Good |
