# Supplementary material for: Longitudinal study of root resorption on incisors caused by impacted maxillary canines—a clinical and cone beam CT assessment
Source: Eur J Orthod. 2024 Oct 16;46(6):cjae052. doi: 10.1093/ejo/cjae052 (PMC11480922; doi:10.1093/ejo/cjae052)
Supplement: cjae052_suppl_Supplementary_Material [file cjae052_suppl_supplementary_material.zip › Appendix_2.docx]

**CLINICAL EXAMINATION**

Probing depth (PD): was measured in millimetres from the gingival margin to the bottom of the gingival pocket on six sites – mesiolingual, lingual, distolingual, distobuccal, buccal, and mesiobuccal using a periodontal probe (Hu-Friedy Perio Probe Qulix-11). Probing depth of ≥4 mm was registered as they were considered pathological [1].

Gingival retraction (GR): was measured mid-buccal and mid-lingual of the teeth and graded as (1) cementoenamel junction not visible, (2) cementoenamel junction and less than 2 mm of root surface visible and (3) cementoenamel junction and 2 mm or more of root surface visible.

Mobility (M): was measured using the “Grace & Smales Mobility Index” [2]: Grade 0: No apparent mobility, Grade 1: Perceptible mobility <1 mm in buccolingual direction, Grade 2: >1 mm but <2 mm, Grade 3: >2 mm or repressibility in the socket.

Ankylosis(A): was diagnosed as normal or high-pitch percussion sound.

Discoloration (DC): was rated as too yellow, too grey, too dark, too light, or not deviant.

Vitality (V): was tested with an electric pulp tester (Pulppen dp2000 digital). The incisors were isolated by a cotton roll and the pulp tester was placed in the middle of the buccal surface of the crown. Patient was asked to inform the operator whenever he or she felt tingling, pain, or any sensation during activation of the electric pulp tester. Teeth were graded as vital or nonvital.

Bonded retainer: registered as present or not.

**REFERENCES**

1. Lang, N.P. and J. Lindhe, *Clinical Periodontology and Implant Dentistry, 6th ed*. 2015: John Wiley & Sons. p. 564.

2. Marya, C., *A Textbook of Public Health Dentistry, 2nd ed*. 2011, Jaypee Brothers Medical Publishers: New Delhi. p. 203.
