# Supplementary material for: Longitudinal study of root resorption on incisors caused by impacted maxillary canines—a clinical and cone beam CT assessment
Source: Eur J Orthod. 2024 Oct 16;46(6):cjae052. doi: 10.1093/ejo/cjae052 (PMC11480922; doi:10.1093/ejo/cjae052)
Supplement: cjae052_suppl_Supplementary_Material [file cjae052_suppl_supplementary_material.zip › cjae052_suppl_Supplementary_Material.docx]

|  | Horizontal root resorption | | | | Vertical root resorption | | |
| --- | --- | --- | --- | --- | --- | --- | --- |
|  | Improved | Unchanged | Worsened | p value | Unchanged | Worsened | p value |
| T0 to intervention IMC | 0.3 ± 0.3 SD | 0.5 ± 0.4 SD | 0.8 ±1.2 SD | 0.207 | 0.5 ± 0.5 SD | 0.5 ± 0.5 SD | 0.755 |
| Treatment duration** | 2.5 ± 0.7 SD | 1.7 ± 1.1 SD | 2.1 ± 0.5 SD | 0.201 | 1.6 ± 1.0 SD | 2.0 ± 1.0 SD | 0.247 |
| Follow-up period*** | 8.4 ± 3.0 SD | 9.1 ± 2.5 SD | 10.4 ± 0.3 SD | 0.577 | 8.7 ± 2.8 SD | 9.3 ± 2.4 SD | 0.469 |

**Supplementary table 1.** The effect of time variables on changes in horizontal and vertical root resorption.

* Time from diagnosing the resorption (T0) until intervention of the impacted maxillary canine (IMC) took place.
** Time with fixed appliance.
*** Time from T0 to follow-up.
